# Round the Hospitals

**Published:** 1918-04-06

**Authors:** 


### ROUND THE HOSPITALS.

There has never been a moment in the course of the war when the message of Easter was more needed. The human wreckage begins to drift back swiftly from the terrible ordeal of battle. Even after skilful and devoted care *en route*, the injured men, briefly classified as "cot cases," move the hearts of those who receive them to a passionate resentment against fate, an overwhelming pity too deep for expression. Custom has stilled some of the poignancy of these arrivals, but the sharp throb of expectancy as the convoy tarries, the stab of grief as these poor fellows are borne in uncomplaining one after another, this never stales from use, and is stifled only when the mind gets too tensely absorbed in labours for the sufferers to register another impression. There are many whose duty has called them close to the battlefield, where visions of carnage, a slaughter beyond anything the world has ever known, have made their brain reel and touched them with an extremity of horror which can never be effaced while life lasts. There are some who declare that the sights they have seen have shattered their faith. How can they believe (such is the argument) in a God who will permit these things? They looked for intervention and found it not. The accumulated weight of war, with its brutal crushing of innocent lives, has blotted out the vision ineffable; the heart goes hungry, the inner light has faded—for a time.

Easter calls us to fix the thoughts less exclusively on the shattered bodies and the bruised and quivering brains, which still shudder from the shock of battle. Of each one it can be said, as of their Master, "his visage was so marred more than any man, and his form more than the sons of men." Each one of these has been wounded for us, bruised for us. The chastisement of our peace rests upon them. With their stripes we are healed. These who lie before us are indeed men of sorrows and acquainted with grief. But in the light of the open grave at Easter-time we learn the true perspective of these hideous happenings. Their elder Brother has gone before them to victory over death, and He calls us all, at this time, away from the contemplation of the broken mortal envelope to fix our vision on the immortal being behind, whom no engine of war can touch. It is on this immortal being, intact, superb, in each maimed form, that nurses need to fix their consciousness, as they strive with tireless courage and patience to bring relief from pain. Easter calls us with a message of transcending hope, and crowned with triumph, away from thoughts of the brief agonies of death to a vision of immortal men unflinching, victorious through their divine birthright. They vanish from our sight, but that army which to-day is thinned by shot and shell still lives on, immortal in the annals of our race, transfigured with the Resurrection glory. Easter brings us the glad certainty of a love sustaining and purifying each myriad personality from which, after a little season of mingled joy and sorrow, the mantle of mortality has been withdrawn. Let us not grieve unduly over the cast garment. Rather let us concentrate this Easter-tide, amidst the torture of suspense, upon the eternal, indestructible personality of our brothers-in-arms as they pass through death to victory, their Master at their head.

The work of forming local centres of the College of Nursing is proceeding rapidly. Birmingham and Leeds have both held meetings during the past ten days with this end in view. At the Leeds meeting, which took place on March 27 at the General Infirmary, a number of matrons from Leeds and Yorkshire discussed the proposition to form a centre for the County of Yorkshire, and it was decided to hold a general meeting at the Philosophical Hall, Park Row, Leeds, on Monday, April 29, at 4 p.m., when the Hon. Sir Arthur Stanley, G.B.E., Chairman of the College, and Miss Rundle, Secretary, will speak. The chair will be taken by Mr. Charles Lupton, Chairman of the Leeds General Infirmary. It is hoped that all matrons of training schools and other institutions in Yorkshire will be present with as many members of their staff as possible, and that those who cannot attend will send their names and addresses to one of the Honorary Secretaries for the Leeds Centre, Miss Lindall, Hospital for Women and Children, Leeds, and Miss Spann, Township Infirmary, Leeds.

It is good news that Leicester is making a move towards forming a local centre of the College of Nursing. A meeting is to be held in the Nurses' Home of the Leicester Royal Infirmary next Tuesday, April 9, to consider how best to form a local branch of the College, and it is to be expected that a strong rally of members of the College, both from Leicester itself and from the neighbourhood, will be the result. The training-school of the Leicester Royal Infirmary has long given a lead in many important particulars, and manifests that it is animated by a truly progressive spirit in identifying itself with the aims of the College of Nursing.

At Manchester the local centre of the College of Nursing is already taking steps to realise one of the chief aspirations which move nurses to combine. A very stimulating course of post-graduate lectures has been arranged to take place at the Lecture Theatre of the Royal Infirmary at 5.30 p.m. The course begins on April 9 and 30 with lectures, illustrated by lantern slides, from Dr. Stopford on the Treatment of Nerve Injuries. On May 14 and 28 Mr. Hey is to lecture on Surgery at the Front and at the Home Hospitals. On June 4

**ROUND THE HOSPITALS—(continued).**

Dr. C. Chisholm lectures on Venereal Disease. The lecture on June 18 will be given by Mr. Telford on Hip Disease and Rickets, at Swinton House, Swinton, and an object-lesson in the modern treatment of these diseases will be available in the Swinton Schools, which will be on view during the afternoon. The last two lectures of the course will be given by Dr. Barclay on July 9 and 23, the subject being Electrical Treatments. Thus a varied and highly interesting programme has been secured. The fee for the course of eight lectures is 5s., or 5s. 6d. for non-members; single lectures 1s. Nurses have long experienced a great need for post-graduate instruction, and we cannot doubt that the attendance will be such as to enable the local committee of the College to continue this admirable branch of work.

---

Their Royal Highnesses Princess Louise, Princess Beatrice, and Princess Arthur of Connaught have signified their intention of being present at the Nurses' Memorial Service at St. Paul's Cathedral next Wednesday, April 10. We have already published directions in regard to applications for seats (The Hospital, March 16, p. 516), and it should be noted that all ticket-holders should be in their places by 1.45 p.m. Copies of the Form of Service, containing the Roll of Honour, can be obtained from the Rev. A. Lombardini, Church of St. Elizabeth, Kensington, W. 8, price 1s. The proceeds will be devoted to the war work of the British Red Cross Society and the Order of St. John of Jerusalem.

---

The *Daily Telegraph* of March 29 has a fine sketch of the proposed memorial to Edith Cavell subscribed for by readers of that journal. It is to take the form of a great granite cross, forty feet in height, to be placed on a site above St. Martin's Church. From this there rises a second cross, with the symbolic figure of Humanity, bearing a child upon her knees. There is a suggestion of a nurse's uniform in the draperies of this figure. Above the head Mercy and Truth fold their wings. The figure of Edith Cavell, carved in white Carrara marble, in her nurse's cloak, will be the prominent central feature of the monument, which Sir George Frampton, R.A., has designed as a labour of love. It is very strange and beautiful to watch the image of this noble nurse becoming transformed throughout the Empire into a symbol of self-sacrifice and healing. From Melbourne this month we learn that the Edith Cavell Memorial amounts already to over £8,000, most of which is to be held in trust for the benefit of military nurses. They are placing a bust of her, designed and executed by Miss Baskerville, in one of the public gardens of the city, with a bas-relief depicting her last hours before execution.

---

Sir George Beatson, K.C.B., K.B.E., chairman of the Scottish Branch of the British Red Cross Society, had some sound advice to give to the volunteers among the staff when he opened the Seafield Auxiliary Hospital a short time back. Seafield House was formerly the residence of that noted bridge-builder, Sir William Arrol, and it has but one defect, considered from the point of view of the present purpose. It is a trifle too splendid even for the reception of heroes, and its priceless woodwork needed some protection in the interests of owners and patients alike. Ayrshire, Sir George Beatson declared, had reason to be proud of the fact that the entire establishments, numbering 6,000 beds, available for Red Cross patients in Scotland were staffed and nursed without any charge upon the Red Cross funds. But, in alluding to misunderstandings which have arisen among V.A.D. workers in certain districts, he told his hearers straight out that these were military hospitals, and that the V.A.D.s, even when accompanied by their Commandant, must remember that they were called on to serve under the matron and that discipline must be maintained. That was the only way in which to secure the harmony so essential in the work of a hospital. These are wholesome words, and lay managers not only in Scotland would do well to lay them to heart. In nearly every case in which friction has arisen it may be traced to jealousy of the matron's proper powers and influence.

---

The public interest in "the treatment of nurses at the Front" is shown by the continuance of the correspondence in the *Times*. Several facts begin to emerge. One is the unswerving loyalty of the whole Nursing Service towards its Chiefs. Another is their uncomplaining endurance of conditions harder than ordinary folk imagined. It is no bad thing that part of the jealously guarded secret of what the nurses have to endure should leak out. Some day there must be searching inquiry as to whether the hardships were in the nature of things, such as could only be conquered by enduring them, or whether they were due to parsimony or incapacity. Should next winter find the world still fast bound in the misery of war, it ought not to be considered impossible to mitigate the severity of the climate in the nurses' sleeping quarters by the adequate provision of oil-stoves, to get them out of tents into huts, and to see that their beds are not exposed to the elements. More than a year ago the Bridgeman Committee insisted on "better accommodation for nurses where it is now inadequate." What has been done in this direction?

---

A very satisfactory account reaches us of the Blanket, Linen, and Work Guild at the Royal Sussex County Hospital. The Guild provides the whole of the household linen, blankets, clothing, rubber goods, etc., needed in the hospital with its 265 available beds, in the nursing home and domestic quarters numbering 116 beds, in the operating theatres, and in the convalescent home at Ditchling. The Guild has recently held its annual display, which was exceptionally full of interest

**ROUND THE HOSPITALS—(*continued*).**

this year, when the sympathies of all thinking people have been called out to work harder than ever for the hospitals. Now is the time for all matrons still unprovided with a Linen Guild to take steps to supply this link in their organisation. The War Depôts have shown how much the amateur can contribute, and longs to contribute, to the relief of suffering. Public effort must not cease when war hospitals are disbanded. It is earnestly to be hoped that some of the War Depôts may become permanent institutions affiliated to the voluntary hospitals throughout the kingdom for the supply of surgical dressings, linen and other necessaries. The best hope for this is to stimulate local effort through the formation of Work Guilds attached to particular hospitals, and of these we know none better organised than the one at Brighton.

---

The Glasgow and West of Scotland Co-operation of Trained Nurses celebrates its twenty-fifth year of existence this month, and since, though it has always had an excellent Sick Fund, it has no Pension Fund, steps have now been taken to form a Benevolent Fund from which grants can be made to supplement the nurses' savings. Nearly all the £3,000 for which appeal was made has been received already; but if this is to be invested the yearly income does not strike us as likely to be adequate, seeing that the staff since the beginning of the century has averaged some 173 members. Theoretically, of course, members of good co-operations are in a favourable position for making provision for the future. In practice so many causes contribute to sap their forces that the proposed grants in aid of older members of the staff will never, we fear, be superfluous.

---

In many hospitals and infirmaries nurses surrender three or four hours a week to the business of food production, and may be seen hard at work trenching, breaking clods, and preparing the ground for planting. In places where allotments are to be had in convenient proximity to the institution, the nurses combine to take up plots, with the added interest of growing what they please and presenting their produce to the charity. The nurses at the Prince of Wales's Hospital, Tottenham, presented 2¼ tons of potatoes to the institution through their labours last year. Probably the idea is capable of much wider extension. After the first shock to the muscles of tackling a hard piece of ground, the labour has a wonderfully invigorating effect, especially upon people whose lives are led indoors. Allotment work is well adapted to the large class who want to help in food production but can only give a fragment of their time. A hundred nurses, giving up an hour every other day to field-labour, are equal to thirty full-time labourers, and if every institution helped, according to its situation and capacity, hundreds of fresh acres could be got under cultivation.

The Exeter District Nursing Association has in view an interesting new departure. The nurses have long required better accommodation, and an emergency appeal for funds made a year ago has enabled the committee to acquire for this purpose a property known as "Dix's Field." The principal rooms in the house are to be fitted up as a maternity home, the need for which has been repeatedly experienced by the nurses in their work. The City Council has agreed to share the expense of equipment and upkeep on condition that two beds are always available for cases recommended by the medical officer of health. The Lying-in Charity also co-operates and has voted £20 for the maintenance of their cases. The experiment will be watched with much interest. Its only danger lies on the financial side, and some considerable skill may be needed to keep the accounts for the nurses' home and the maternity home entirely separate as they ought to be. But if this is done there can be no objection to running the two concerns side by side. It is now generally recognised that in-patient accommodation is essential to any complete scheme of infant and maternity welfare, and the more closely this form of treatment is linked up with the nurses who know the mothers and are trusted by them, the more likely it is that women will avail themselves of it. There is a proposal to relieve the minds of the mothers by sending home-helps to their houses to look after their children and keep things straight in their enforced absence, and this will no doubt help forward the success of a very well thought-out scheme.

---

At the thirteenth annual meeting of the Association for Promoting the Training and Supply of Midwives, held a short time ago, it was stated that the Association received during the past year 204 applications for training, as compared with 280 in 1916. Miss Amy Hughes read a paper on "Proposals for a State-aided Service of Midwifery," urging that nothing below 25s. a case could be considered adequate remuneration for the midwife. We fear it is bad policy to attempt to force up the fee above the usual doctor's fee for the delivery prevailing throughout the country. The Association would do good service if it would collect information as to the fees charged in the towns and villages by midwives provided out of voluntary subscriptions. It is quite useless for the ordinary midwife to compete with midwives supported by these societies, and the first step to a reform in fees is to realise that the clients of benevolent nursing societies are not exclusively selected from the "poor," but are for the most part fairly prosperous working people well able to pay a proper fee.

---

**☛ It is our invariable practice to pay for all items of news which we may publish. The minimum payment is 5s. Paragraphs of about twenty lines in length—that is to say, of 150 words or less—are preferred. Contributions should be addressed to The Editor, The Hospital, 28 and 29 Southampton Street, Strand, London, W.C. 2, and, to be in time for the current week's issue, should reach him by the first post on Mondays.**

